# Supplementary material for: Immune Heterogeneity and Epistasis Explain Punctuated Evolution of SARS-CoV-2
Source: medRxiv. 2022 Jul 29:2022.07.27.22278129. Preprint. [Version 1] doi: 10.1101/2022.07.27.22278129 (PMC9387145; doi:10.1101/2022.07.27.22278129)
Supplement: 1 [file NIHPP2022.07.27.22278129V1-supplement-1.pdf]

medRxiv preprint doi: https://doi.org/10.1101/2022.07.27.22278129; this version posted July 29, 2022. The copyright holder for this preprint (which was not certified by peer review) is the author/funder, who has granted medRxiv a license to display the preprint in perpetuity.
It is made available under a CC-BY 4.0 International license .

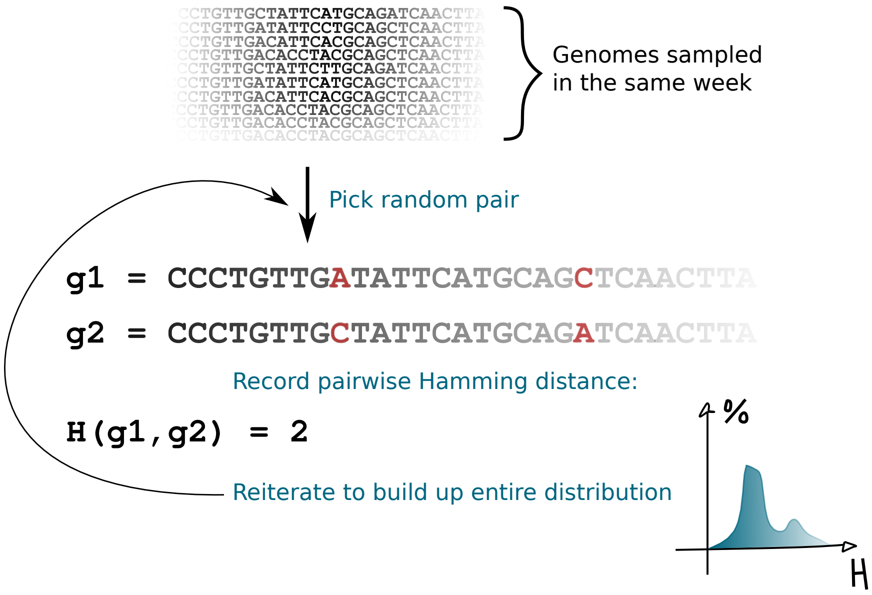

Figure 1–Figure supplement 1. Data analysis workflow. To generate the Hamming distribution for a given point in time, all sequences sampled within a week-long window starting on the given day are pooled. Then, pairs of sequences are repeatedly selected at random from this sequence pool, and the pairwise Hamming distance (number of sites which differ) is computed. All the computed Hamming distances are then pooled and a distribution (histogram) is generated.

medRxiv preprint doi: https://doi.org/10.1101/2022.07.27.22278129; this version posted July 29, 2022. The copyright holder for this preprint (which was not certified by peer review) is the author/funder, who has granted medRxiv a license to display the preprint in perpetuity.
It is made available under a CC-BY 4.0 International license .

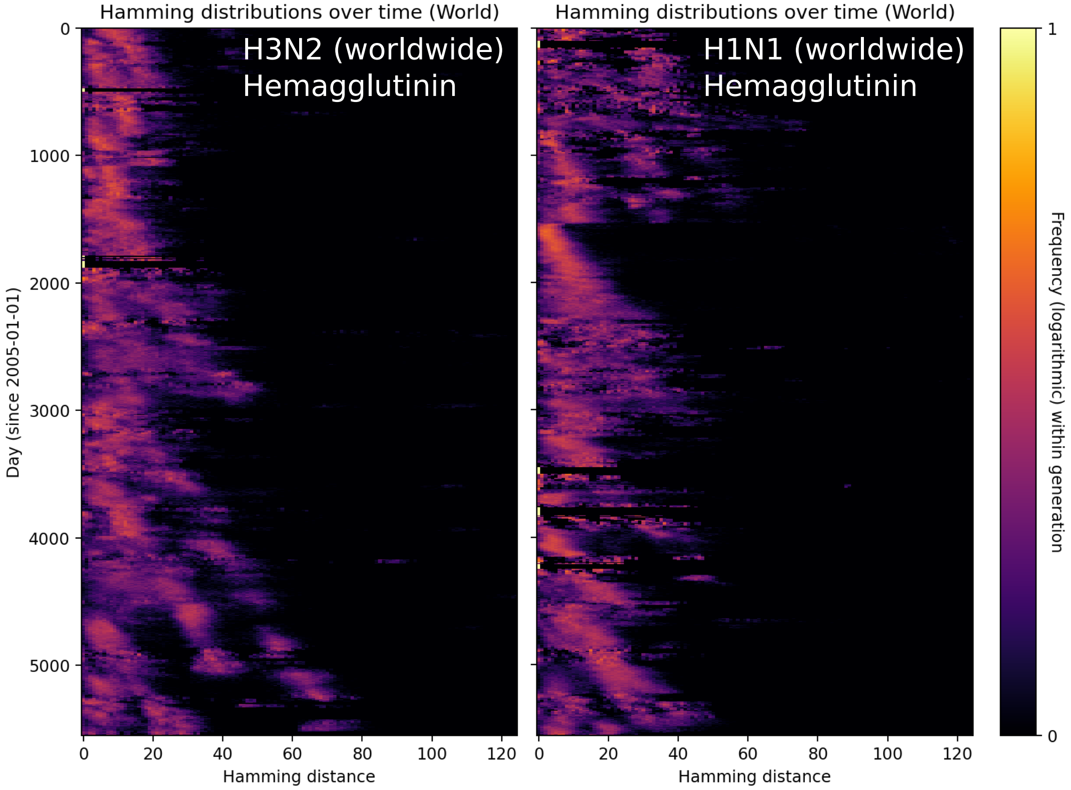

Figure 1–Figure supplement 2. Hamming distributions for influenza H3N2 and H1N1. With influenza, the amount of genomic surveillance data is much more limited and the temporal Hamming distributions are much less well-defined. In order to have enough data for each time point, a sampling window of 30 days was used here, as opposed to the 7 days used for SARS-CoV-2 in the main text.

medRxiv preprint doi: https://doi.org/10.1101/2022.07.27.22278129; this version posted July 29, 2022. The copyright holder for this preprint
(which was not certified by peer review) is the author/funder, who has granted medRxiv a license to display the preprint in perpetuity.
It is made available under a CC-BY 4.0 International license .

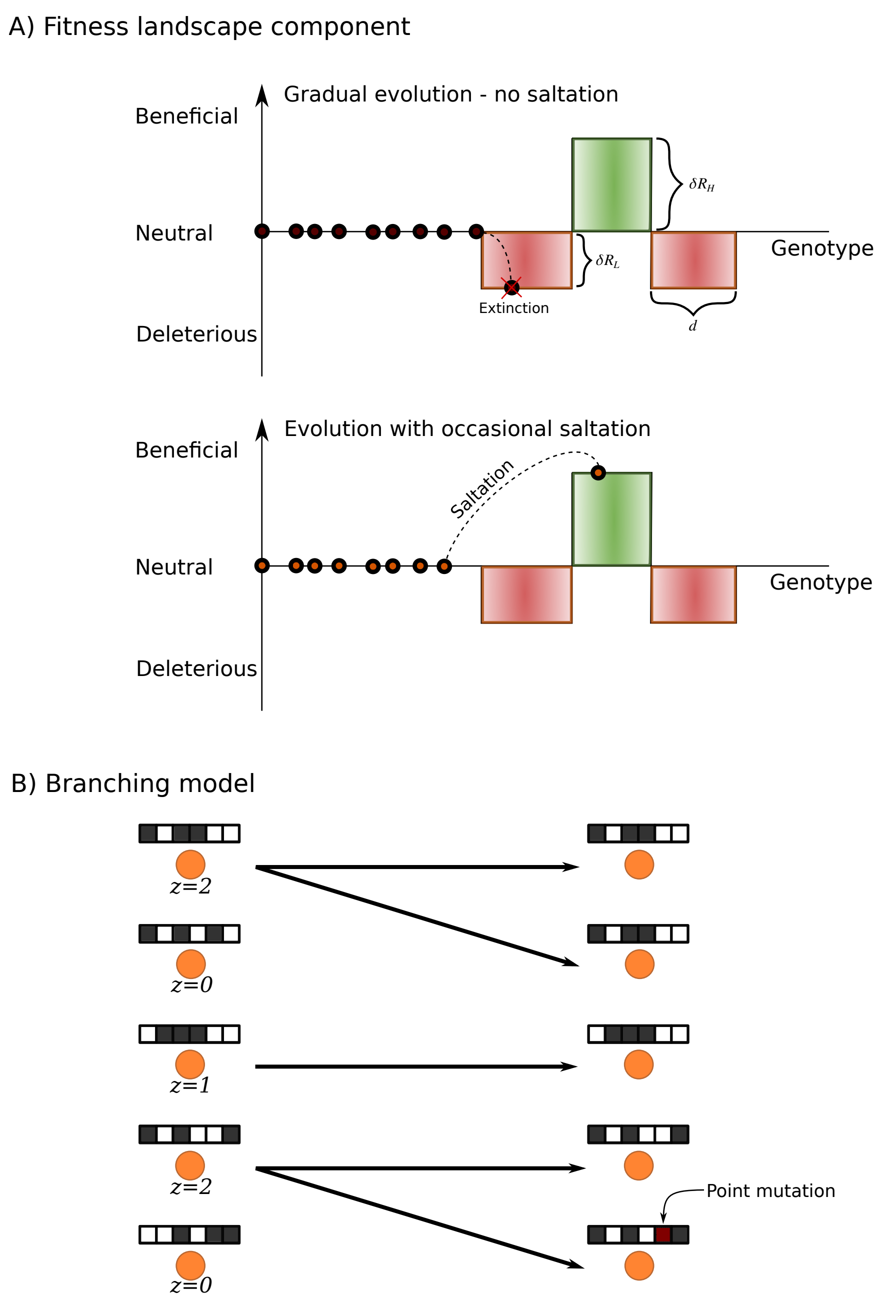

Figure 3–Figure supplement 1. Model schematics. A) The fitness landscape and epistasis components of the model. The majority of the fitness landscape is assumed neutral. In the case of gradual evolution devoid of saltation (top), the pathogen performs a random walk in this neutral space until it hits upon a deleterious configuration. As a model of sign epistasis, beneficial configurations are surrounded by deleterious ones. In the case of gradual evolution, the deleterious regions are unlikely to be traversed before the lineage dies out. However, in the case of saltational evolution (bottom), several point mutations may occasionally happen in the same genome within the same generation, leading to a jump which can enable the pathogen to bypass a deleterious region. Note that this is only a 1-dimensional conceptual representation of a highly multidimensional fitness landscape. **B)** In each generation of the branching model, each individual stochastically infects z new individuals. Upon infection, the pathogen genome (depicted as a string of black and white squares) is transferred. Occasionally a point mutation will occur, as indicated in the lower right genome. In the case of saltation (see panel A), multiple such point mutations can occur within the same genome in the same generation.

medRxiv preprint doi: https://doi.org/10.1101/2022.07.27.22278129; this version posted July 29, 2022. The copyright holder for this preprint (which was not certified by peer review) is the author/funder, who has granted medRxiv a license to display the preprint in perpetuity.
It is made available under a CC-BY 4.0 International license .

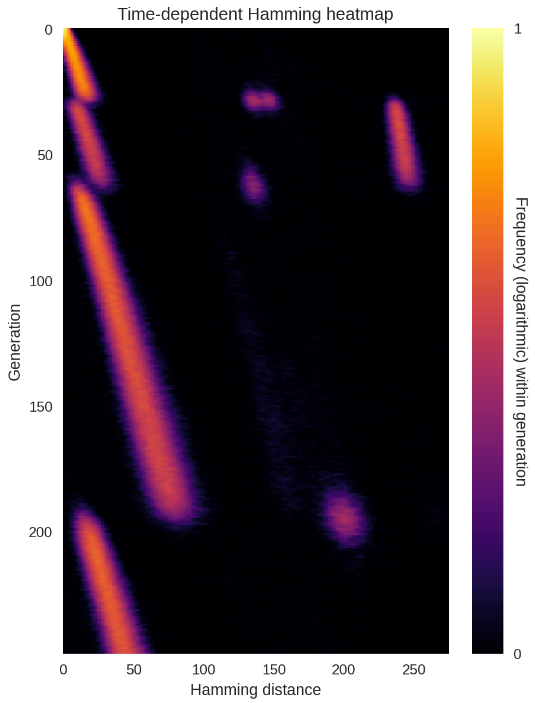

Figure 3–Figure supplement 2. Temporary coexistence of two equally fit variants.

medRxiv preprint doi: https://doi.org/10.1101/2022.07.27.22278129; this version posted July 29, 2022. The copyright holder for this preprint (which was not certified by peer review) is the author/funder, who has granted medRxiv a license to display the preprint in perpetuity.
It is made available under a CC-BY 4.0 International license .

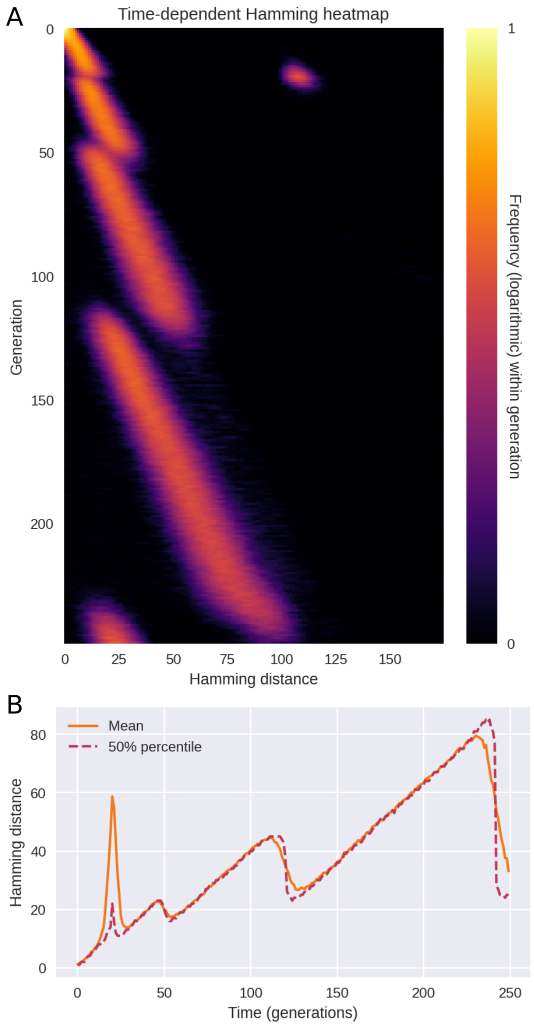

Figure 4–Figure supplement 1. Saltational evolution in the absence of sign epistasis. When saltational evolution is allowed, but epistasis is absent or very weak, a mixture of qualitatively different transitions occur. Some resemble the diversity spikes seen in Figure 3, but more commonly transitions will involve a gradual, linear increase in diversity followed by a collapse, as seen in Figure 4. **A)** Time evolution of the Hamming distance distribution. For each generation indicated on the vertical axis, the colour encodes the histogram of Hamming distances between genomes within that generation. **B)** Time evolution of the mean and median Hamming distance between genomes present in any given generation of the model simulation. In these simulations, $\delta R_L = 0$ (no epistasis) while saltations were of typical size $\mu_1 = 150$.

medRxiv preprint doi: https://doi.org/10.1101/2022.07.27.22278129; this version posted July 29, 2022. The copyright holder for this preprint
(which was not certified by peer review) is the author/funder, who has granted medRxiv a license to display the preprint in perpetuity.
It is made available under a CC-BY 4.0 International license .

725

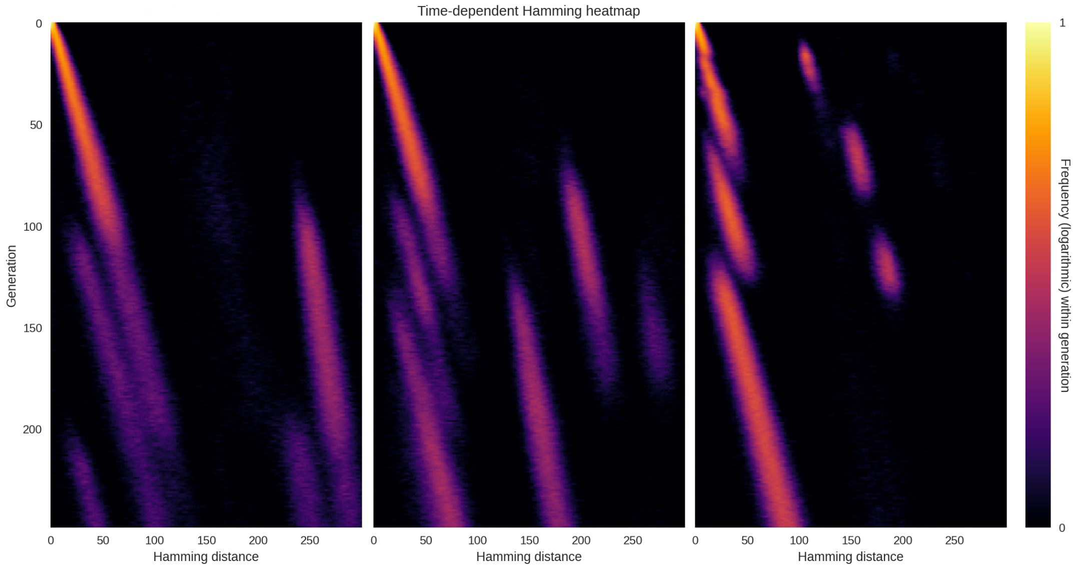

Figure 6–Figure supplement 1. Spatial structure leads to prolonged transition. Here we simulate the same SIRS dynamics as in Figure 6, but in a metapopulation consisting of three subpopulations. The within-population transmission rate $T_{ii} \approx 1$ ($i \in \{1, 2, 3\}$) is assumed much greater than the between-population transmission rate T_{ij} (with $j = i \pm 1$). **(A)** With inter-population transmission rate $\beta_{i,i\pm1} = 0$, mutations never spread from one population to another and coexistence of variants with different fitness can last indefinitely. **(B)** With an inter-population transmission rate of 10^{-4}, transitions are severely prolonged but coexistence of variants with different fitness values does not last indefinitely. **(C)** At an inter-population transmission rate of 10^{-3}, transitions are only moderately prolonged compared to the non-spatial dynamics of Figure 6.
